# Reducing pressurised metered-dose inhaler prescriptions for asthma to reduce carbon emissions: a qualitative study of healthcare professional perspectives

**DOI:** 10.3399/BJGPO.2024.0208

**Published:** 2025-12-19

**Authors:** Lauren Franklin, Christian Mallen, Helen Twohig

**Affiliations:** 1 School of Medicine, Keele University, Newcastle-under-Lyme, UK

**Keywords:** asthma, inhalers, primary health care, qualitative research

## Abstract

**Background:**

Prescribing of pressurised metered-dose inhalers (pMDIs) is a key NHS carbon hotspot and reducing the number of these devices prescribed will help achieve NHS net zero targets.

**Aim:**

To explore primary healthcare professionals’ perspectives on reducing the prescribing of pMDIs for people with asthma to reduce associated carbon emissions.

**Design & setting:**

Qualitative study of healthcare professionals (GPs, practice nurses [PNs], and clinical pharmacists [CPs]) working in general practice in England.

**Method:**

Eighteen semi-structured interviews were conducted with healthcare professionals. Participants were recruited through professional networks and using snowball sampling. Topic guides were developed to explore participant perspectives, concerns, and motivations.

**Results:**

Eight GPs, six PNs, and four CPs were interviewed. Results are presented over two topics. The first explores factors influencing inhaler device choice and discusses the following themes: patient-centred care; bias and assumptions; clinician confidence and knowledge; and status quo of asthma care. The second topic identifies facilitators and barriers for prescribing fewer pMDIs through the following themes: understanding; attitudes to change; confidence in dry powder inhalers (DPIs); attitudes to change; engagement with sustainable prescribing; and system drivers.

**Conclusion:**

Interlinking personal, consultation, and external factors influence which inhaler device is prescribed for patients with asthma. There are considerable actionable barriers to implementing carbon-conscious prescribing, many of which would improve the quality of asthma care.

## How this fits in

This is the first qualitative study set in UK primary care to explore factors that influence healthcare professionals’ decisions when prescribing inhaler devices and facilitators and barriers to reduction of pMDI use. Survey data suggest that the medication required, the disease profile, and the patient’s previous experiences are the greatest factors affecting device choice but there is little exploratory qualitative data available.^1,2^ This study provides the foundations for developing interventions to promote prescribing of lower carbon inhalers.

## Introduction

NHS England aims to be carbon net zero for the emissions it directly controls by 2040. Primary care is responsible for 23% of NHS emissions and needs to decarbonise for the overall NHS target to be reached.^3–5^ Pressurised metered-dose inhalers (pMDIs), which are commonly prescribed for asthma, account for 3% of total NHS carbon emissions and 13% of primary care’s emissions at point of use, making it a key target for emissions reduction.^3^

Asthma is the commonest chronic respiratory condition in the UK, with 5.4 million people receiving treatment.^6^ Inhaled medications are the mainstay of asthma treatment and can be prescribed in different device types, including pMDIs, soft mist inhalers, and dry powder inhalers (DPIs). pMDIs use gas propellants to deliver the medication, which include potent greenhouse gases. DPIs do not contain these gases and therefore have a much lower carbon footprint. For most people with asthma, pMDIs and DPIs are equally efficacious.^7,8^ pMDIs are the most prescribed type of inhaler in the UK, accounting for 70% of total inhaler prescriptions; this is much higher than the proportion prescribed in other European countries.^1,9,10^

Initiatives and resources have been introduced to aid healthcare professionals to reduce pMDI prescribing, such as the inclusion of financial incentives in the 2022–2023 Investment and Impact Fund (IIF), National Institute for Health and Care Excellence (NICE) decision-making tools, and the low carbon asthma care toolkit.^11–14^ However, there is little research about clinician perceptions of the issues and what might influence change in practice.

The aim of this study was to explore healthcare professionals’ decision-making processes when choosing which inhaler to prescribe for asthma and their perspectives on reducing the proportion of pMDIs prescribed to reduce primary care’s carbon footprint.

## Method

### Study design

Qualitative study using semi-structured interviews.

### Patient and public involvement and engagement (PPIE)

A meeting was held with three people with asthma during the development stage of the study to explore their perspectives on the issues to inform the topic guides.

### Participant recruitment

GPs, practice nurses (PNs), and clinical pharmacists (CPs) employed at primary care practices in England with an active role in the care of patients with asthma were recruited, initially through advertising among the team’s professional networks. Potential participants were sent an information sheet and consent form. Further participants were recruited through snowball sampling, with purposeful recruitment used to ensure variation in clinician role, sex, and clinical experience. Participants were offered a £50 shopping voucher as compensation.

### Data collection

Interviews were conducted via Microsoft Teams by LF. Informed consent was obtained in advance and reconfirmed at the start of the interview. Two topic guides were used for the interviews, one for GPs and PNs and one for CPs to reflect their different roles in asthma management. The research team developed both topic guides, with input from healthcare professionals and the PPIE group, and modified iteratively, concurrently with interviews and data analysis.

Transcripts were obtained using the automated feature of Microsoft Teams, checked for accuracy against the original recording, anonymised, and imported into NVivo (version 12). Interviews continued until saturation of themes was reached in the overall participant population as well as within each professional group.

### Analysis

The data were analysed using reflexive thematic analysis principles outlined by Braun and Clarke with a predominantly inductive approach.^15^ LF coded all transcripts and HT coded a subset of transcripts to sense-check ideas and explore different interpretations of the data. Codes and subsequent themes were discussed and developed iteratively throughout the analysis and a reflexive diary was maintained throughout. Analysis and interpretation of the data is framed through the lens of the researchers, comprising two practising GPs and one medical student at the time of the study. After 18 interviews, no new codes were identified, and data saturation had been reached.

## Results

Eighteen participants (eight GPs, six PNs, and four CPs) were interviewed between December 2022 and May 2023. Ages ranged from 24–54 years, and number of years qualified ranged from 1–28 years. The practice Index of Multiple Deprivation (IMD) covered the full range 1–10, with higher representation from practices in more deprived areas. There were two practices from which two participants were recruited. The average duration of interview was 32 minutes (range 22–40 minutes). Table 1 summarises participant demographics.

**Table 1.** Participant demographics

Participant	Age, years	Sex	Role	Years qualified	IMD (/10)
GP1	37	M	GP (partner)	9	1
GP2	36	F	GP (partner)	6	2
GP3	38	F	GP (partner)	8	5
GP4	40	F	GP (salaried)	12	2
GP5	36	M	GP (salaried)	7	4
GP6	34	M	GP (salaried)	2.5	4
GP7	47	M	GP (salaried)	18	1
GP8	50	M	GP (partner)	19	5
PN1	51	F	PN	13	7
PN2	54	F	PN	28	2
PN3	45	F	PN	18	1
PN4	40	F	PN	4	2
PN5	51	F	PN	17	7
PN6	38	F	PN	11	3
CP1	24	F	CP	1	10
CP2	37	M	CP	1	5
CP3	54	F	CP	11	1
CP4	27	M	CP	4	5

CP = clinical pharmacist. PN = practice nurse. IMD = Index of Multiple Deprivation

**Figure 1.**
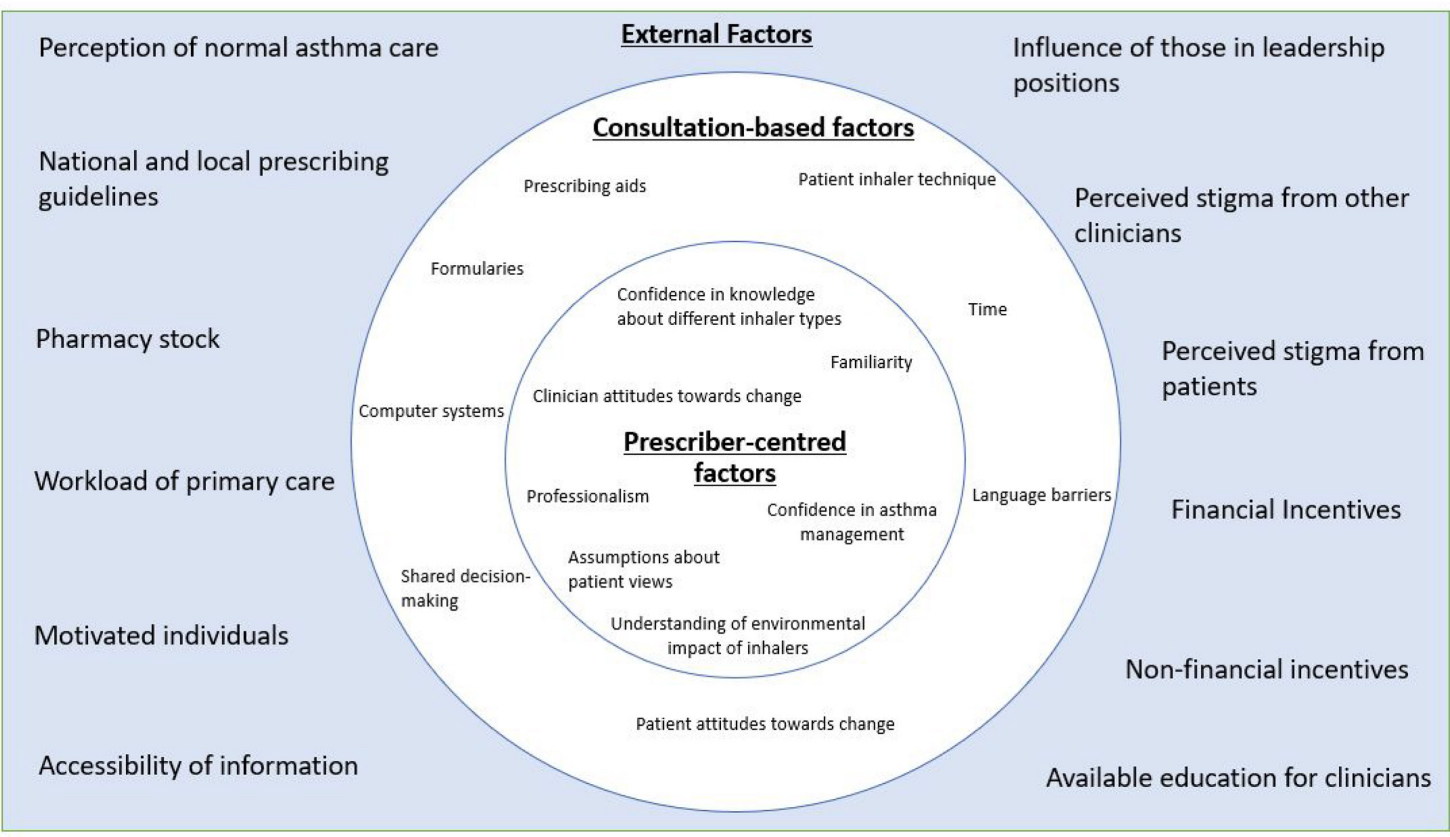
Summary of the factors that influence inhaler device choice

Findings are divided into two topics: ‘Decision making in inhaler prescribing’ and ‘Reducing the proportion of pMDIs prescribed’. Figure 1 summarises the identified prescriber, consultation, and external factors affecting inhaler prescribing.

### Decision making in inhaler prescribing

The following four themes were identified: patient-centred care; bias and assumptions; clinician confidence and knowledge; and status quo of asthma care.

### Theme 1: Patient-centred care

Participants described their prescribing decisions as being predominantly determined by individual patient factors. The concept of the ‘best’ inhaler for an individual incorporated clinical effectiveness, patient preference, and ability to use the device correctly:

*'So I would look at the history to see if and what inhalers* [they] *are already on and I would work out what’s happening with them. If they’re stable, if they're uncontrolled, if they're using too much of something, too little of something. And base my decision on what I feel will be better for them. So it’s always a clinical decision based on what would suit the patient best, but I often explain to patients with inhalers, it’s often a trial-and-error process and it’s finding the right one for them.'* (PN6)

*'Our care should be patient focused and should be, you know, about ensuring that it’s the right inhaler for the patient. Yet of course there is a drive to say it should be all about the green agenda and everything else. And, whilst I'm 100% supportive of that, that can't be to the detriment of patient care.'* (GP7)

Patient-specific factors commonly identified to influence device choice included practical considerations, such as dexterity and willingness to use a spacer, and the sensation of using the treatment:

*'So you wanna have one that’s quite easy to put in your pocket and carry around. And sometimes that’s how I might perhaps sell it. And you know, that’s the benefit of powder because they've literally just got that little, you know, one to carry around their pocket, not with the spacer and that kind of thing. And yeah, a lot of people don't wanna carry spacers around with them as well.'* (PN5)

*'Lots of my patients are preferring the DPI, mostly as they find it easier to just click and click and go. There’s no coordination required.'* (GP1)

*'Cause some patients really like the fact that they can taste it and then other patients really, really hate the fact that they can taste it.'* (PN3)

Social factors, including financial concerns and family experience, also influenced inhaler choice:

*'... the area we work in are really deprived, so if they're on three different inhalers and they're not affording them or they're sharing them around the house, which often happens, you've got a family that are using each other’s inhalers. I'll try and get them on just a combined all-in-one inhaler so it’s cheaper for them as well.'* (PN6)

*'Obviously asthma has quite strong family links. Usually mum or dads got it and you're more likely I think to get it drilled home rather than teach parent a new technique to show to kid, whereas you could improve parent’s technique of their own inhaler and then they're gonna show the kid better.'* (CP4)

Some participants described changing inhaler type to match a patient’s existing technique rather than trying to change someone’s technique to match their device:

*'Most people, even who have given an aerosol, aren't doing it right. Their technique is a dry powder, so I tend to now look at someone’s technique more, basing it on whether or not I'm gonna switch them to a dry powder.'* (PN1)

### Theme 2: Bias and assumptions

Assumptions based on demographic factors affected inhaler choice. Participants were less likely to prescribe DPIs for older patients or to consider changing their inhaler type. It was also assumed that older patients would be less motivated by environmental concerns:

*'They* [older adults] *often can't grasp new inhalers. They can't put their mouth right. They can't do this. They can't do that, and they can't often get that big, short, big breath that you need for that. They're more slow and steady, and I think they would be harder to educate as well as their ability to do it.'* (PN1)

*'But actually if you're using four salbutamol inhalers a year, that is making a huge contribution to your carbon footprint, and I think a lot of younger people will certainly respond to that. I don't know how people in their 80s and 90s would respond to it.'* (GP2)

Deprivation, ethnicity, and language barriers also affected the likelihood of practitioners suggesting a change to someone’s inhaler or discussing lower carbon options:

*'Deprived areas also not bothered — they have more pressing matters to deal with.'* (CP4)

*'We have two sites. We have one which is very white working class and we have one which is massively ethnically diverse and we have to use interpreters all the time and totally honestly, I wouldn’t even think about doing it there because usually the interpreters are on the phone.'* (PN3)

### Theme 3: Clinician confidence and knowledge

Participants demonstrated a lack of confidence in their knowledge about inhaler devices and technique, with GPs considered by themselves and others to have the least knowledge:

*'7% of healthcare professionals understand exactly and can demonstrate how to use an inhaler. That’s not good enough. So we actually need to be taught properly ourselves, so we need to do effective training, which then will mean our patients are trained.'* (CP3)

*'I bet if — I'm just making so many horrid assumptions about our wonderful GPs, but they are brilliant — but I think I feel like if I ask them actually tell me how you take, even an MDI they wouldn't have a clue about. And I don't think they'd know the difference between how you do an MDI or DPI.'* (PN3)

*'I'm not entirely convinced I know the difference in terms of all the different devices, 'cause I often don't initiate things.'* (GP6)

Familiarity was a key factor in inhaler device choice with many participants viewing pMDIs as the ‘*normal*’ or ‘*standard*’ inhaler. Reliever and preventer inhalers were often described by the corresponding colour of widely used pMDIs, such as ‘*blue and brown*’:

*'The kind of almost standard that were coming into, if that makes sense, what the established like … precedent which you know has been before is MDI, which is you know the staple within the UK. That’s what the majority of prescribers and patients are familiar with. And then that’s often what is initially started.'* (CP1)

*'To be honest, most inhalers tend to be started by a doctor. And they always do the standard blue and brown.'* (PN1)

Education was deemed important in improving confidence around inhalers and their environmental impacts through inclusion within continuing professional development (CPD), practice training, and undergraduate curricula. However, there were concerns around the need for additional training being overwhelming:

*'You have to change it from the beginning, from these young students when they're coming through becoming GPs and becoming doctors, if they're not doing this first line, nobody’s gonna change.'* (PN1)

*'There’s only so many education meetings you can do on inhalers before people switch off before you even started.'* (GP1)

### Theme 4: Status quo of asthma care

In addition to participants’ candidness about their own lack of confidence in some areas of asthma management, a sense that there are high levels of suboptimal asthma control arose from the interviews.

Poor inhaler technique was acknowledged to be a common issue:

*'So I will always start with whether they're actually using them, getting to actually tell me what they're using and when they're using it cause inevitably it’s wrong.'* (PN3)

*'I mean it’s horrendous sometimes when you see what they're actually doing. I mean one man sprayed it on his neck.'* (PN4)

Other common issues were acceptance and normalisation of symptoms:

*'I find asthmatics normalise their condition so they could, you know day-to-day just have this residual wheeze and like they are in the morning and things like that and they say "oh, that’s because I'm asthmatic."'* (CP1)

Overreliance on short-acting β_2_-agonists (SABA) was felt to be difficult to tackle in many cases owing to ingrained habits. This has implications for the ability to reduce pMDI prescribing given that the majority of SABAs are prescribed in pMDI form:

*'I have a lady who’s been using three* [salbutamol inhalers] *in a month last week.* […] *for some of them like that lady, it’s an addiction. She can't not have it* [...] *trying to take that off somebody who’s had it for 20 years is extremely difficult.'* (PN6)

When considering how to implement changes in inhaler prescribing, important context can be provided by understanding primary care clinicians' perspectives regarding the scale of the challenge of improving asthma care.

### Reducing the proportion of MDIs prescribed

The following five themes were identified: understanding; confidence in DPIs; attitudes to change; engagement with sustainable prescribing; and system drivers.

### Theme 5: Understanding

All participants demonstrated an awareness that inhalers have an environmental impact; however, the level of understanding varied:

*'I can't work out if it’s something to do with gases and I don't really understand it.'* (PN3)

*'I say the words but I don't really know what I mean. I say "it reduces the carbon footprint and it’s good for the environment."'* (CP3)

Lack of knowledge limited participants’ confidence to suggest inhaler changes to patients:

*'If you're not educated on it and a patient says, "well why are you moving to me to that?". Because I've been told to. It’s not a good enough answer, is it?'* (PN6)

Poor understanding was linked to lack of training, clinician age, poor information availability, and intangibility of some key concepts:

*'I do think it’s a real generational thing. I'm in my 50s and you know, it’s a modern thing to talk about. Do I really understand it? No, I don't.* […] *Maybe the younger generations understand it more because they're more into it.'* (CP3)

*'I think it’s hard to see. I've got no idea what a ton of CO_2_ looks like or what having a ton less of CO_2_ in the atmosphere actually means.'* (PN2)

Participants demonstrating higher levels of understanding tended to equate carbon emission of inhalers to car miles, showing the power of the widely used infographic:^16^

*‘*[discussing recent PCN teaching] *I think the slide that hit home the most was the one, I'm sure you've seen it, which is like "1 Ventolin allows you to drive from* [city] *to London" and then 1 dry inhaler was like 4 miles and everyone’s like "wow".’* (GP01)

### Theme 6: Confidence in DPIs

Some concerns were expressed about safety of DPIs in particular clinical situations and for some participants this was a considerable barrier to prescribing these inhalers:

*'I've heard, at least anecdotally, I haven't seen any patient myself, you know, where the switch has been made and because it’s a dry powder and maybe isn't appropriate for that patient with like a poor inspiratory effort, then there’s that’s exacerbating their asthma and then running the risk like proper exacerbation, you know, risk of harm, risk of hospital admission.'* (GP5)

*'You can't do a short sharp breath when you're having an asthma attack, but that is what I would say that there* [are] *some concerns that way around.'* (PN1)

Counter to these concerns, the widespread use of DPIs in other countries was cited by some participants as providing them with reassurance regarding safety:

*'You know when if you read about studies in other countries, like in Sweden and things where you know the majority of their salbutamol inhalers are DPIs, then it does give me confidence that, Oh no, it can actually … it’s like it’s quite safe and it is been done. So why can't we do it?'* (CP1)

### Theme 7: Attitudes to change

There was general acknowledgement that changing prescribing patterns is difficult, whatever the driver:

*'Well getting anyone, especially a doctor in the NHS, to change is very hard and that’s not specific to inhalers or anything, so that’s probably the main barrier, just like people don't like change. They know what they know and kind of stick to old guidelines.'* (GP5)

Barriers to change were closely linked to the themes identified when exploring decision making. The tendency to prefer to stick to familiar inhalers and a perception that patients would not be amenable to change was evident:

*'If I'm looking for a steroid inhaler, I do tend to use Clenil. Just as it was the* [location of the practice] *policy, it was their first-line choice for ages and I think that’s still in my head.'* (GP4)

*'We know that clinicians are comfortable with prescribing what they know.'* (GP8)

*'People don't like change and I think that’s the problem with asthma. People think they know the condition and they don't like change.'* (CP2)

Lack of time and clinician burnout impacts on capacity to develop new knowledge and skills, and on capacity to make changes during consultations:

*'Something has to give. And sometimes sticking with the status quo is the thing that makes life able to be tolerable and to stop your GP from having to work 14–15 hours a day.'* (GP2)

*'There’s not necessarily that time to be able to do it and change people over and making sure that, you know, if you do change them over that they're using the inhaler correctly and confidently.'* (PN5)

*'I feel quite relieved when I have a patient already on a dry powder inhaler that I don't have to have that conversation.'* (CP3)

### Theme 8: Engagement with sustainable prescribing

Some clinicians feared stigma if they openly discussed environmental considerations within prescribing decision making:

*'Some people roll their eyes and go "ohh, you know the environment" and you know those people who are always gonna be sceptical.'* (PN4)

Opinions differed on whether it was the role of a clinician to advocate for lower-carbon alternatives and whether there could be tension between what was best for the environment and what was best for an individual:

*'What I think they will expect from the doctor and their expectations of like a kind of like unbiased professional, you know with only their interests at heart. I think as soon as you start saying anything about like, well, this is a bit cheaper, this is a bit better for the environment, I think you sometimes lose a bit of trust and they think that you're like trying to con them into getting something that’s inferior.'* (GP5)

*'And it’s a no-brainer really. Like they’re the same price or cheaper, and a better for the environment and the patient outcomes are the same, so.'* (GP1)

Discussing the impact of health care on the environment was seen to be difficult and time-intensive and some clinicians felt they were poorly equipped to have these discussions:

*'It can sometimes be tricky to have those conversations. I think the environmental side of things is slightly easier to sell than just a cost and yeah, you certainly do come up against resistance if you're trying to change someone without sort of justifiable reason.'* (GP6)

Having informed and motivated individuals in a practice team was identified as an enabler but the need for systematic change and support from integrated care boards (ICBs) and primary care networks (PCNs) was highlighted:

*'That kind of strongly leadership at least has got rid of a bit of that stigma and kind of normalised it a bit and started to change that culture.'* (GP5)

*'I think it has to be the whole area has to be onboard with it and so within a primary care network, all the practices and GPs and nurses need to be upskilled at the same time and start this across the board and in agreement to which guidelines you use* […] *everyone needs to be singing from the same hymn sheet.'* (CP2)

### Theme 9: System drivers

Despite the tendency of participants to focus on decisions made in the context of the clinician–patient dyad, the limitations of individual actions were acknowledged:

*'I always find as an individual GP even now as a partner you still need a system to be changing rather than just an individual.'* (GP2)

Participants identified financial incentives, such as the IIF, as key drivers in raising awareness and encouraging practices to prescribe more lower-carbon inhalers:

*'There is like no way in hell that the whole PCN would have switched … from Ventolin to Salamol if it wasn't for that financial reward.'* (CP1)

Potential issues, including increased workload, difficulty in reaching thresholds, and prescribing DPIs inappropriately to reach these targets were recognised. Financial incentives were also perceived to impact GPs, particularly partners, more than other members of the primary care team:

*'Paying GPs to do something equals getting it done. I must be quite perfectly and completely honest with that. And because if they think there’s gonna be money at the end of it, they'll do it. And they'll actively encourage the nurses that are prescribing to do it as well.'* (PN2)

Other incentives identified included the Green Impact for Health Toolkit awards and inclusion of prescribing targets in the Quality and Outcomes Framework (QOF).

External resources used to support prescribing, including local and national guidelines, formularies and websites, such as RightBreathe,^17^ were identified as ways to promote low-carbon alternatives:

*'One of the problems we always have with sort of asthma is well, which guideline do I use? And I think if there is clear and unequivocal combined guidance produced regarding the use of DPI preferentially over pMDI, then I think that would put us in a much better position.'* (GP7)

Computer pop-ups and asthma review templates that included environmental considerations also prompted behaviour change:

*'If you want the truth, it’s because it’s included on the template.'* (PN1)

*'On the Ardens template, on SystmOne, it kind of tells you what devices there are and has a little picture of them and has their environmental impact on there as well. And I'm like quite keen on that so I always try to choose the lowest environmental impact yeah first.'* (GP5)

In addition to workload and time pressures that emerged in earlier themes, pharmacy stock was identified as a further external constraint that prevented change in practice:

*'Probably wrongly, I would then shy away from* [DPIs] *because I think like it’s gonna be hard, the pharmacies are less likely to have it in, the patient’s gonna need to wait each month for pharmacy to order it in.'* (GP4)

## Discussion

### Summary

Decision making around inhaler prescribing is complex. Clinicians describe basing decisions on what is ‘*best*’ for the patient but underlying this is a hidden layer of influencing factors including clinician knowledge and confidence, values, system pressures, and assumptions based on patient demographics.

Barriers to prescribing fewer pMDIs include clinician inertia, lack of understanding about the carbon footprint of inhalers, and concerns about safety of DPIs in particular groups or clinical situations. Ingrained cultural assumptions and time and workload pressures also inhibit change in prescribing practice.

### Strengths and limitations

Inclusion of participants from different disciplines, with analysis that compared and contrasted opinions between groups, has provided a rich understanding of the context in which changes to inhaler prescribing need to be made. This study sample varied by age, sex, experience within primary care, and deprivation of GP practice but we acknowledge that participation bias may have influenced the findings.

The fact that the interviews were framed through the lens of the environmental impact of inhalers is important in the interpretation of the findings. The scope of the interviews did not include broader aspects of asthma care and the discussion about inhalers was focused on device rather than medication choice. We recognise that this is a somewhat artificial distinction as management decisions are always taken in a wider context but the aim of this study was specifically to explore perspectives on the net zero targets as a driver for change.

This study was focused on NHS England and, while strategies and targets differ across the devolved nations (Scotland, Wales, and Northern Ireland), barriers within day-to-day clinical practice are likely comparable. While there was representation from all over England, the pragmatic recruitment strategy adopted meant that half of the participants were from the same broad geographical area and local prescribing policies or educational initiatives may have impacted the findings.

### Comparison with existing literature

Previous studies have reported that type of medication, familiarity, and perceived ease of use were important factors in clinicians’ decisions about which inhaler to prescribe.^2,18–21^ Our findings emphasise the importance of familiarity with particular devices and uncover some of the reasons for clinical inertia, which include personal factors, such as knowledge and confidence and relationships with colleagues and patients, and system factors such as time pressures and pharmacy stock. These are important considerations when considering how to encourage and enable clinicians to change their prescribing patterns.

While the focus of this study was on inhaler device choice, broader challenges in asthma management cannot be ignored. These provide the context against which change has to happen.

Inequalities in asthma care based on ethnicity and deprivation were identified in this study, which align with well-reported disparities in patient outcomes across ethnic and socioeconomic groups.^22–26^ It is evident that unjust cultural assumptions affect clinical practice and may contribute to inequalities in care. Unacceptable rates of poor asthma control, worldwide and in the UK, have been repeatedly reported.^27,28^ There are many contributing environmental and healthcare system factors but one repeatedly identified clinical factor is suboptimal use of inhaled therapies, underuse of anti-inflammatory maintenance therapies, overuse of SABA inhalers, and poor inhaler technique. Our findings suggest a lack of professional knowledge and confidence around inhalers, particularly among GPs, but also a perception that patient understanding of asthma is often inadequate.

While our study focused on perspectives on lower-carbon inhaler prescribing, the findings serve to illustrate the scale of the challenge faced by those providing front-line asthma care with regard to optimising inhaled therapy. Poorly controlled asthma is associated with increased morbidity and mortality but also much higher carbon emissions (as a result of increased SABA use, increased healthcare contacts, and hospital admissions).^29,30^ Improving the overall quality of asthma care therefore has the dual benefits of improving health outcomes for individuals and reducing carbon emissions.

One approach that exemplifies this win-win opportunity is greater use of anti-inflammatory reliever (AIR) therapy, which can be given as needed in mild asthma or in maintenance and reliever therapy (MART) regimens. This approach uses a single combined inhaled corticosteroid (ICS)-formoterol inhaler, which is usually a dry powder device. It has been shown to improve symptom control and reduce exacerbations, and international guidelines now recommend AIR therapy first line (Table 2). If the UK were to widely implement this strategy, it could improve asthma outcomes and significantly reduce the number of pMDIs prescribed.^31^

**Table 2.** 

Changing context of asthma guidelines
This study was carried out before the release of the new BTS and SIGN guidance on the management of asthma.^32^ The new guidance provides a welcome shift towards the use of AIR and MART regimens first line for people aged >12 years, and recommends that no-one should be prescribed a SABA without concomitant ICS prescription. Implementation of this approach is likely to considerably reduce the numbers of pMDI devices prescribed. However, the findings from our study highlight the complexity of introducing changes to practice and are therefore salient for those considering how to implement the new guidance.

AIR = anti-inflammatory reliever. BTS = British Thoracic Society. ICS = inhaled corticosteroids. MART = maintenance and reliever therapy. pMDI = pressurised metered-dose inhaler. SABA = short-acting β_2_-agonists

### Implications for research and practice

This study has identified factors that influence inhaler device choice from the perspective of primary healthcare professionals, and provided insight into barriers and facilitators to reducing associated carbon emissions. Further research to explore patient and public views is needed, but findings from this study could inform development and testing of strategies or interventions to promote reduction of pMDI use while improving the quality of asthma care.

Given the complexity of organisational structures within primary care, it will take multiple different approaches, at different levels, to facilitate widespread change. Education and training of professionals and changes to clinical guidelines may help address knowledge and confidence gaps but addressing wider system pressures, re-introducing financial incentives, updating formularies and templates, and facilitating leadership at national and local level are all also required to achieve the significant reductions in pMDI prescribing that will enable net zero targets to be reached.

## Data

The dataset relied on in this article is available from the corresponding author on reasonable request.
